# Hopium or empowering hope? A meta-analysis of hope and climate engagement

**DOI:** 10.3389/fpsyg.2023.1139427

**Published:** 2023-08-15

**Authors:** Nathaniel Geiger, Timothy Dwyer, Janet K. Swim

**Affiliations:** ^1^Indiana University Bloomington, Bloomington, IN, United States; ^2^The Pennsylvania State University (PSU), University Park, PA, United States

**Keywords:** hope, climate engagement, policy support, behavior, discussion, information seeking, meta-analysis, systematic review

## Abstract

Researchers are increasingly examining whether hope can motivate action on climate change, or conversely, whether it might demotivate such action. We present a meta-analysis (*k* = 46) of quantitative studies examining the relationships between measures and manipulations of hope with climate engagement. On average, *measured* hope was associated with greater climate engagement (*r* = 0.18); however, this effect differed based on the target of hope. Hope regarding the possibility of respondents taking action was particularly strongly associated with greater engagement (*r* = 0.40), while in contrast, hope grounded in climate change not being a problem was associated with less engagement (*r* = −0.40). Hope in response to climate change generally, and domain-general hope, were only weakly associated with greater engagement (*r*s = 0.13, 0.20). On average, hope *manipulations* fostered increased engagement, though the increase was small (Cohen’s *d* = 0.08). Subgroup analyses suggested two promising types of hope manipulations warranting future research: *personal efficacy* (*k* = 2, *d* = 0.18) and *in-depth* (*k* = 2, *d* = 0.49). In contrast, messages suggesting societal efficacy (i.e., providing a sense of possibility that climate change could be addressed) did not significantly or substantially boost (nor discourage) engagement (*d* = 0.05), and status quo-framed messages (i.e., messages highlighting that environmental conditions could stay the same if climate action is taken) had a marginally significant negative effect on engagement (*d* = −0.18). After excluding a single outlier, the extent to which manipulations increased hope were not correlated with increases in climate engagement, suggesting the possibility that hope might be incidental to the success of some manipulations rather than a necessary component for promoting engagement. Overall, our meta-analysis does not suggest that increasing hope decreases climate engagement, with the possible exceptions of denial hope and status quo framed messages. Conversely, however, results provide partial yet inconclusive evidence for the hypothesis that increasing hope increases climate engagement. Given the existing published literature, we argue that future researchers should consider study designs that align with theoretical perspectives on how hope promotes climate engagement (e.g., longitudinal designs) and also consider directly assessing populations of interest (e.g., climate activists).

## Introduction

Adequately addressing climate change requires widespread public engagement and mobilization to demand and cooperatively enact the substantial societal changes needed to address this existential threat. A rapidly emerging body of research examines the motivational power of hope when working toward a more climate-friendly future. Two recently published papers help synthesize this expanding research. Park et al. (2020) review the literature on hope in environmental conservation (broadly, not climate change-specific) and reconcile variations in definitions of hope used within this literature. Ojala’s (2022) brief narrative review provides insights into which measures of hope relate to climate engagement. We expand upon these two initial explorations by conducting a meta-analysis of the quantitative literature examining the relationship between hope and climate change engagement. By doing so, we build upon the insights yielded by these previous reviews and improve the field’s understanding of whether hope relates to climate engagement, and if so, when and how this might be the case.

## An overview of hope and climate change engagement

Hope is a complex cognitive-emotional-motivational state that attunes individuals to the possibility of desirable future outcomes (Ortony et al., 1990; Peterson and Seligman, 2004). The emotional components of hope reflect an anticipatory state often experienced as positively valenced (i.e., a pleasant state). Hope is also commonly conceptualized in terms of its cognitive and motivational components that tend to accompany the emotional experiences of hope (Malle, 2004; Peterson and Seligman, 2004; Geiger et al., 2019a). Some scholars have defined and measured hope in terms of a cognitive-motivational axis rather than an emotional experience (Snyder, 2002), while others argue that the motivational components central to Snyder’s definition of hope are not properly part of the experience of *hope* and better fit under the distinct concept of *efficacy* (Van Zomeren et al., 2019). Despite differences, all definitions share a common focus on hope as a future-oriented state that orients people toward imagining positive futures (Fernando et al., 2018; Kantenbacher et al., 2022). This conceptualization has led many climate advocates and researchers to consider whether or how hope might motivate climate engagement.

It is also possible that different psychological processes might underlie connections between hope and different forms of climate engagement, potentially leading to hope promoting some forms of engagement more than others. For example, if hope is closely associated with personal efficacy (i.e., the perception that one can personally contribute to making a difference; Magaletta and Oliver, 1999; Feldman and Kubota, 2015), because personal efficacy is a robust predictor of climate action (Doherty and Webler, 2016; Geiger et al., 2017), but not necessarily policy support, hope might be more strongly associated with behaviors than with policy support. Conversely, if hope is more closely associated with societal efficacy (i.e., the perception that it is possible for society to address climate change), hope might be more strongly associated with policy support than behaviors. As another example, hope might uniquely increase interest in learning more about climate change if it sustains people to feel comfortable seeking out information about climate change (Ojala, 2016) or if people prefer messages about climate change with positive emotional valence to other messages (Skurka et al., 2022). Hope could also have a different impact on actual behavior than on behavioral intentions or willingness to engage in behavior; Brosch (2021) argues that emotions directly impact motivational tendencies toward climate action but only indirectly impact action.

The target in response to which hope is elicited could also influence its effects on climate engagement. Van Zomeren et al. (2019) propose that hope often helps boost coping with stressful events but—depending on boundary conditions—can foster two different types of coping styles with different effects on climate engagement. They argue that hope can foster either (1) problem-focused coping, motivating individuals to act on behalf of the possibility of a better future, or (2) emotion-focused coping, helping individuals to reduce stressor-induced-negative emotions and thereby not altering or even reducing the urgency to act. This latter type of coping is sometimes colloquially referred to as *hopium*, a portmanteau of “hope” and “opium” reflecting the possibility for hope to exert a pleasant yet sedating, demotivational effect (e.g., see Martin, 2021). Other researchers have speculated on how the target of hope could influence which of these responses is likely. Geiger et al. (2021b) propose that feeling hopeful about the possibility of acting on climate change is likely to motivate climate action more than feeling hopeful about climate change more generally (also see Swim et al., 2023). Conversely, Ojala (2012a,b) demonstrate that those reporting hope based on the perception that climate action is not needed are less likely to take action.

It is also possible that different types of hope manipulations might be differentially effective at promoting action on climate change. For example, hope manipulations may be particularly effective when they also promote a sense of efficacy (Cohen-Chen and Van Zomeren, 2018), while those that promote a sense that climate change is not a serious problem may decrease engagement (Ojala, 2012a). Additionally, hope manipulations’ effectiveness at increasing engagement may increase as they exert greater increases in hope. Conversely, even if increasing hope leads to increased engagement, hope manipulations may be ineffective at promoting engagement if they do not successfully increase hope.

## Present research

We present a meta-analysis examining hope as a predictor of climate change engagement. A meta-analysis is a type of systematic review that quantitatively aggregates and synthesizes statistical findings from previous work (Siddaway et al., 2019). Through this meta-analysis we hope to integrate previous scholarly work in this domain, providing a knowledge base that later work can build upon.

Our study explores four key research questions. First, we conduct an omnibus test of eligible research to examine whether hope is related to climate engagement overall (RQ1). Second, we test whether effect sizes vary for different outcome measures representing different types of engagement with climate change (e.g., private vs. public behaviors) and different methods of assessing engagement (e.g., self-reported behaviors vs. behavioral intentions; RQ2). Third, we examine how different studies have measured hope and test whether the relationship between hope and climate engagement differs based on how hope is measured (RQ3). Fourth, we examine how different studies have manipulated hope and test whether some types of manipulations are more effective than others (RQ4).

## Methods

### Literature search and inclusion criteria

We conducted a systematic literature review on previous quantitative research that empirically assessed the relationship between hope and climate change engagement outcomes. We used methodologies based on recommended best practices for conducting systematic reviews in general (Moher et al., 2009) and in psychology (Siddaway et al., 2019). Studies were considered eligible for inclusion if they were quantitative studies involving a survey component that (a) measured and/or manipulated hope, (b) measured a climate change-specific engagement outcome, and (c) we could ascertain the relationship between the two. We included all measures that were termed “hope” by the authors. We included hope manipulations that were either (a) described as a “hope appeal,” “hope-inducing message,” or similar phrase by the authors, and/or (b) empirically demonstrated to increase hope relative to a control condition (e.g., an efficacy-promoting message that empirically increased hope). We operationalize *climate engagement* as climate action (measured or self-reported; including talking about climate change), intentions, willingness, or motivation to engage in climate action, support or acceptance of climate policies, and information seeking. We were not interested in outcomes related to other emotions, opinions, or beliefs about climate change. Similarly, we were not interested in studies that examined other environmental issues not directly connected to climate change. In Fall 2022, we conducted a systematic search of the scientific literature using the databases PSYCInfo and WebofScience, using the following search terms:

- “hope” (in the abstract or title)
- “climate” OR “global warming”
- behav^*^ OR act^*^ OR talk^*^ OR discuss^*^ OR accep^*^ OR “response” OR respond^*^ OR learn^*^ OR engage^*^ OR convers^*^ OR support^*^ OR inten^*^ OR motivat^*^

Our initial search revealed many papers that did not report quantitative results. Because we were interested in quantitative research, we restricted our search to peer-reviewed journal articles, dissertations, and book chapters from edited volumes,^1^ which we anticipated would capture the vast majority of quantitative research that had received independent peer or expert review. We did not examine “gray” or unpublished literature because we anticipated that the quality of unpublished research might be highly variable. Before conducting the search, we compiled a list of 20 articles relevant to the project that we were familiar with and believed a search should capture; we found that all 20 were included in the search results, suggesting that our search methodology adequately represented the relevant literature.

**Figure 1:**
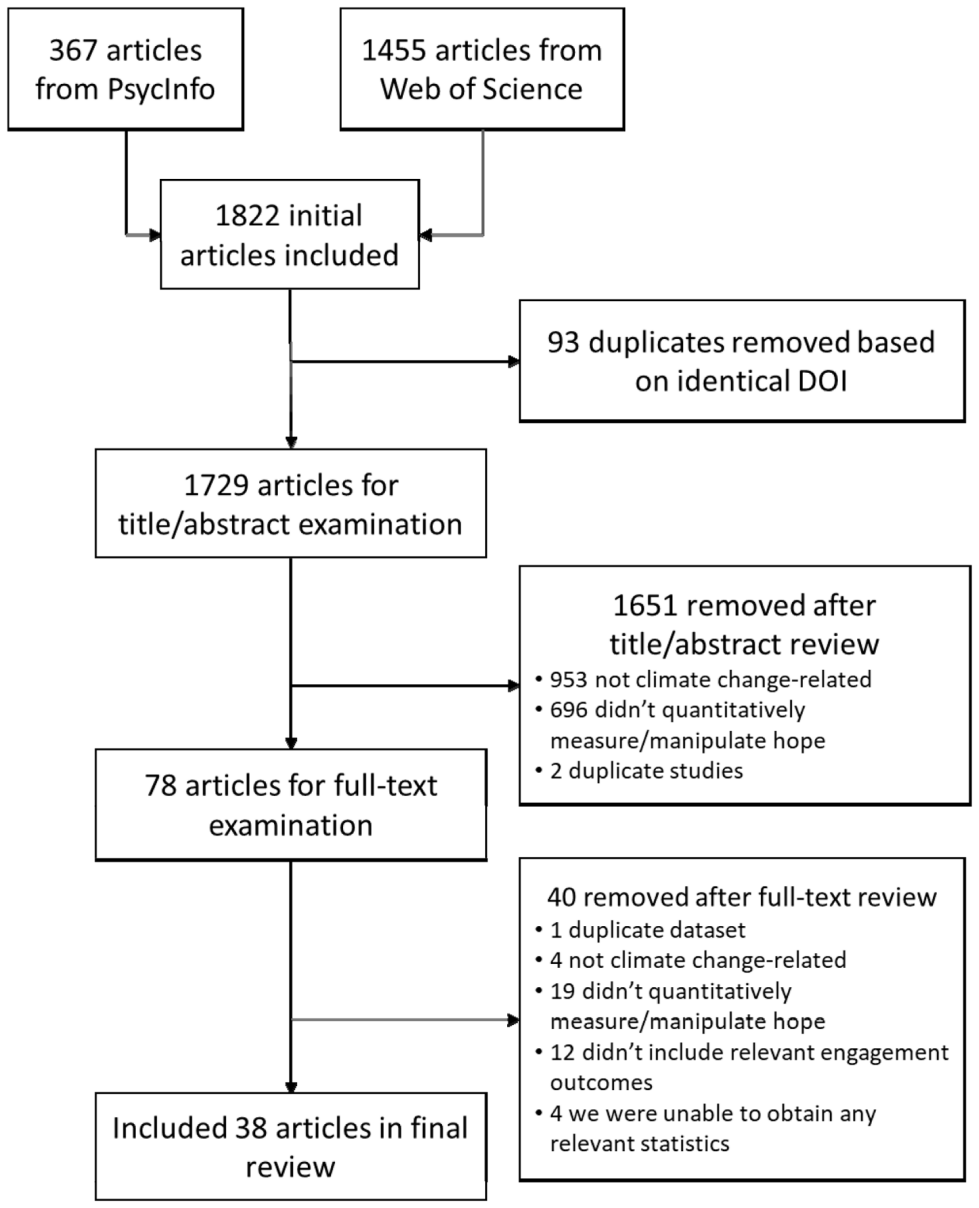
Preferred reporting items for systematic reviews and meta-analyses (PRISMA) diagram.

As shown in Figure 1, we identified 1,455 results from WebofScience and 367 results from PsycInfo. Ninety-three duplicates were identified based on identical DOI, leaving a total of 1,729 articles that the second author screened for eligibility by examining the abstract and title. This title/abstract screening led to 1,651 exclusions. The remaining 78 articles were examined in a full-text review led by the second author, with extensive discussion with the other two authors; decisions about whether to include or exclude articles were made by consensus.^2^ Through this process, we found 21 articles that were potentially suitable for inclusion but the authors did not include condition means/differences between conditions, a correlation matrix, and/or access to raw data in the manuscript; the first author emailed the 18 corresponding authors of these 21 articles (some people had authored multiple articles) asking them to share appropriate statistics or raw data; then sent a follow-up email 1–2 weeks later if they had not yet responded. Twelve of these 18 authors provided the missing information in response to our queries, providing us full information for an additional 13 papers. For four of the other eight papers, we could extract partial information useful for the meta-analysis from the paper itself (e.g., effects of a manipulation but not the zero-order correlation between measured hope and an outcome); we included this partial information in the meta-analysis. For the remaining four papers, we did not have any information suitable for inclusion in the meta-analysis and thus excluded the article entirely. In total, 40 articles were removed through the full-text review process. This left 38 articles to be included in our review and meta-analysis. Details of inclusion and exclusion procedures, as well as a full list of articles that were excluded, are documented at https://osf.io/3wku4/.

## Results

### Overview of studies

Table 1 summarizes the 38 articles, including a total of 46 studies, examined in this meta-analysis. Twenty-two studies were purely correlational (i.e., cross-sectional surveys that tested correlations between measured hope and outcome measures), and 24 included a relevant experimental component (i.e., tested the impact of a hope manipulation on outcomes). Most studies that we classified as experimental were between-participants with random assignments to groups; the exception were two studies we classified as experimental (Geiger et al., 2019a, main study; Rooney-Varga et al., 2018) that did not have control groups and instead compared the treatment groups post-treatment to themselves, pre-treatment (see the section “Study designs,” below, for more details). Some experimental studies tested multiple manipulations; in total, 33 hope manipulations were included in the present meta-analysis. For 17 of the 24 experimental studies, we were also able to obtain correlations between a measurement of hope (e.g., some studies measured hope after the manipulation) and engagement outcomes. Including these correlations yielded a total of 39 studies with correlations between hope and climate engagement. Spreadsheets with statistics that we extracted from each article and details about classifications are available at https://osf.io/3wku4/.

**Table 1:** Studies included in the systematic review.

Authors	Study #(s)	Sample/Population	Outcome type(s)	Outcome measure(s)	Hope measure(s)	Hope manipulation(s)
Armbruster et al. (2022)	1	University students (Canada)	Policy support		Message	Societal efficacy, Status quo frame (factorial)
Arpan et al. (2018)	1	Adult quota (United States)	Policy support		Message^*^	Importance
Baldwin et al. (2023)	1	High school (Australia)	Public	Self-report	Goal	No
Bilandzic et al. (2017)	1	Adult quota (Germany)	Policy support		Message	Status quo frame
Bukchin and Kerret (2020)	1	Farmers (Nepal)	Adaptation	Self-report	Domain-general	No
Bukchin-Peles and Kerret (2021)	1	Farmers (Senegal)	Adaptation	Self-report	Domain-general	No
Bury et al. (2020)	1,2	Mixed college student and adult (Australia)	Policy support (S1, S2)		Actions (S1, S2), Goal (S1)	No
Carmona-Moya et al. (2021)	2	Adult convenience (Spain)	Public	Self-report	Goal	No
Carroll-Monteil (2023)	1	Snowball sample (United Kingdom start)	Private	Abstract	Message	No
Chadwick (2010)	1	University students (United States)	Private	Intentions	Message	Combined
Chadwick (2015)	1	University students (United States)	Private	Intentions	Message	Three importance, one societal efficacy (factorial)
Chu and Yang (2019)	1	Mturk workers (United States)	Private, Policy support	Intentions	Climate change^*^	Importance
Ettinger et al. (2021)	1	Mturk workers (United States)	Public, Private	Abstract	Message	Combined
Feldman and Hart (2016)	1	Adult quota (United States)	Public	Intentions	Message	Two Personal Efficacy, one Societal Efficacy (four levels)
Feldman and Hart (2018)	1	Adult quota (United States)	Public	Intentions, Measured	Message	Three societal efficacy (partial factorial)
Feldman and Hart (2021)	1	Adult quota (United States)	Policy support; Public	Intentions	Climate change	Two societal efficacy (factorial)
Finnegan (2023)	1	High school students (United Kingdom)	Public	Self-report	Goal	No
Furlong and Vignoles (2021)	1	Adults w/ Extinction Rebellion (United Kingdom)	Public	Self-report, Intentions	Goal	No
Geiger et al. (2019a)	1,2	S1: College students (United States); S2: Environmental educators (United States)	Public (S1, S2)	Abstract (S1), Self-report (S2)	Actions (S1, S2)	In-depth (S2 only)
Geiger et al. (2021a)	1	Mturk workers (United States)	Private	Intentions	Climate change	No
Geiger et al. (2021b)	1	Zoo & aquarium visitors (United States)	Public	Intentions	Actions	No
Hornsey and Fielding (2016)	1,2	S1: Adults (United States, United Kingdom, Australia); S2: Adults (United States)	Private (S1, S2), Public (S1)	Abstract (S1, S2)	Climate change (S1), Message (S2)	Societal efficacy (S2 only)
Lee et al. (2017)	1	Convenience (mostly university affiliates; Taiwan)	Private	Abstract	No^*^	Status Quo frame
Maartensson and Loi (2022)	1	Not reported	Private	Self-report	Goal	No
Marlon et al. (2019)	2	Nationally representative adults (United States)	Public, Policy support	Intentions	Actions; Denial	No
Ojala (2012a)	1	High school students (Sweden)	Private	Self-report	Denial, Goal	No
Ojala (2015)	1	High school students (Sweden)	Private, Public	Self-report, Intentions	Denial, goal	No
Park (2020)	1	College students (United States)	Policy support, Private, and Public	Intentions	No^*^	Combined
Pleeging et al. (2021)	1	Panel of adults (Netherlands)	Policy support		Denial, Climate change, Domain-general	No
Rolfe-Redding (2020)	1,2	S1: Nationally representative adults (United States); S2: Quota (Not reported)	Policy support (S1, S2), Public (S1, S2), and Information-seeking (S2)	Self-report (S1), Intentions (S2)	Climate change (S1, S2)	Combined (S2 only)
Rooney-Varga et al. (2018)	1	Participants playing World Climate Simulation^**^	Public	Intentions	Climate change	In-depth
Russell and Ashkanasy (2021)	1–3	S1/2: Undergrad business students (Australia); S3: Working adult convenience (Australia)	Private (S1, S2), Information-Seeking (S3)	Measured (S1–S3)	No	Societal efficacy (S1–S3)
Smith and Leiserowitz (2014)	1	Natl. reprs. Adults (United States)	Policy support		Climate Change	No
Swim and Bloodhart (2015)	1	Mturk workers (United States)	Public	Measured	Goal	Importance
Swim and Fraser (2013)	1	Environmental educators (United States)	Public	Self-report	Actions	No
Thomas et al. (2022)	1	Adult convenience (Canada)	Policy support		Denial	No
van Zomeren et al. (2019)	1–3	Mturk workers (United States)	Public	Intentions	Goal (S1–S3)	Two societal efficacy (S1, factorial), societal efficacy (S2, S3)
Wang and Chen (2022)	1	Middle school students (China)	Private, Information-seeking	Self-report	No^*^	Combined

^*^Hope was measured, but we were unable to obtain relationships. ^**^Study used an array of samples from numerous countries, including in the Global North and South. All policy support measures were abstract; for simplicity, these are not listed here. See https://osf.io/3wku4/ for additional details.

**Figure 2:**
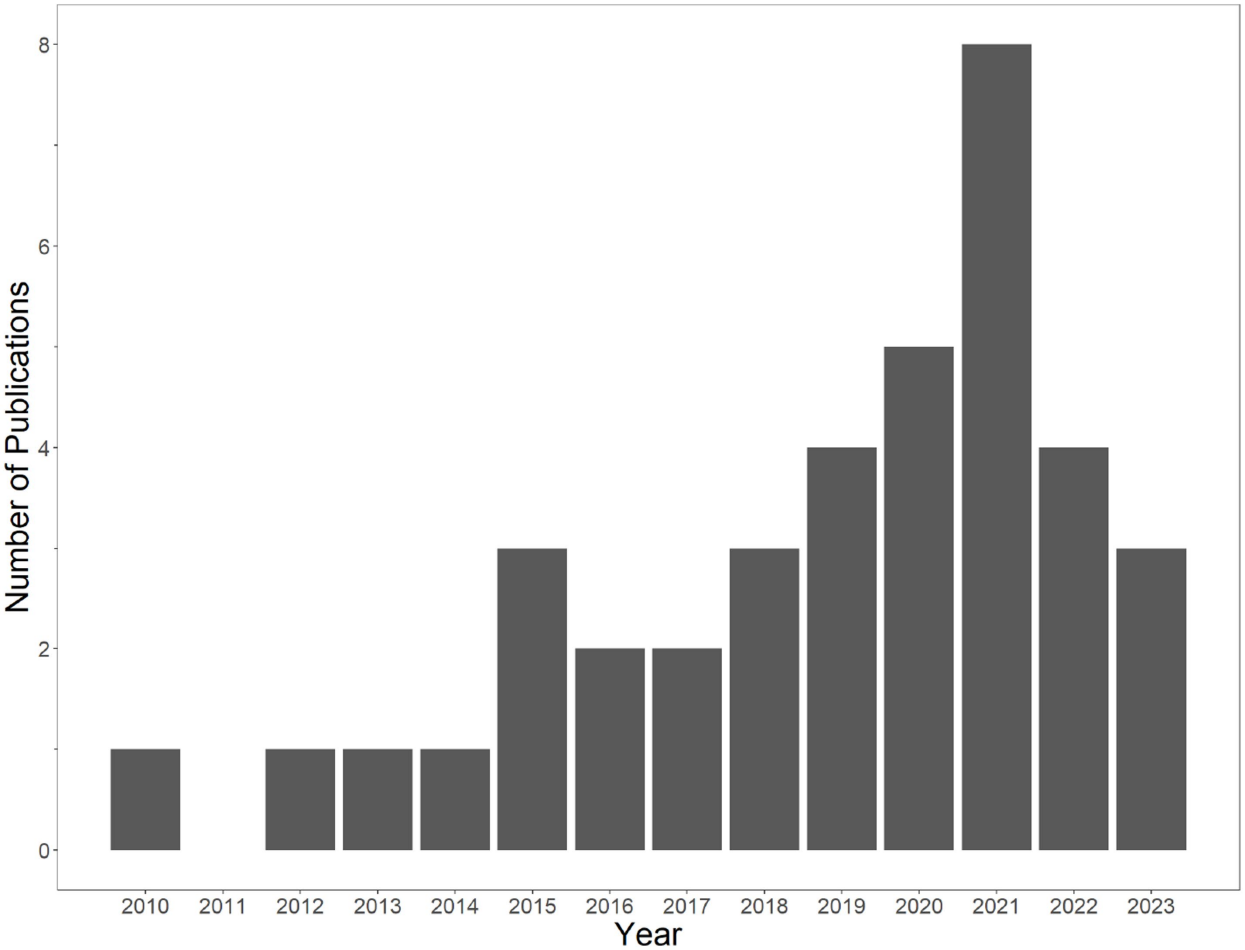
Articles included in meta-analysis by year of publication. Articles in press or not yet assigned to an issue as of June 2023 were marked as 2023. The search was conducted in September 2022, meaning that our review did not include articles published in late 2022 or 2023.

Figure 2 illustrates that all papers identified for inclusion were published in 2010 or later, with the number increasing over time. The earliest article included, from 2010, was a dissertation (Chadwick, 2010). The earliest article included that was published in a peer-reviewed journal was in 2012 (Ojala, 2012a). There were eight papers included from 2021 (the last complete year of the systematic literature review).

#### Participants and populations represented

As shown in Table 1, studies included in this review examined a range of populations, including several countries (mainly in the Global North), with participants ranging from middle-school students to adults. Most studies sampled participants from the general public or students. These populations are largely at least somewhat concerned about climate change but do not tend to be very engaged with the topic (Maibach et al., 2011; Leiserowitz et al., 2021). A few focused on more specific subpopulations: climate activists who are already engaged (*k* = 1) and farmers in the global South (*k* = 2), or environmental educators (*k* = 2). However, because of the limited number of studies that examined these subpopulations, we do not have enough studies to examine whether and how results might generalize to more engaged groups (e.g., climate activists) or other subpopulations of possible interest, such as businesspeople, political leaders, or farmers.

#### Journals represented

Table 2 lists journals with multiple articles published that were included in the present meta-analysis. As can be seen from the table, many articles came from a journal where they were the only eligible article from that journal, and a few journals had published several articles that were included in the present meta-analysis. Of the journals with multiple articles, many were interdisciplinary (e.g., *Climatic Change*, *Global Environmental Change, Sustainability,* and *Risk Analysis*), and other journals represented environmental education (*Environmental Education Research*), psychology (*Journal of Environmental Psychology*), and communication (*Science Communication*). Notably, given the topic, *Environmental Communication* had not published many articles included in this meta-analysis: they had published a single early article on the topic (Swim and Bloodhart, 2015) but no other articles since. Examining the table also reveals that most journals that had published multiple articles had at least some articles with studies involving manipulations of hope. The one exception was *Environmental Education Research,* which had published five articles involving only cross-sectional correlational data (many of these papers also included other information not included in the present meta-analysis, such as qualitative data).

**Table 2:** Journals with multiple published articles included in meta-analysis.

Journal	# articles (# studies)	# Experimental studies	% articles with ≥ 1 experimental study
*Environmental Education Research*	5 (5)	0	0
*Climatic Change*	4 (5)	3	75%
*Sustainability*	4 (6)	5	75%
*Science Communication*	3 (3)	3	100%
*Global Environmental Change*	2 (5)	4	100%
*Journal of Environmental Psychology*	2 (2)	1	50%
*Risk Analysis*	2 (2)	1	50%
*Dissertations only*	2 (3)	2	100%
*All other journals*	14 (15)	5	36%
Total	38	24	53%

Van Zomeren et al. (2019) and Russell and Ashkanasy (2021) each had three experimental studies.

#### Study designs

Studies used a variety of different designs. For purposes of understanding most common study designs, we examined similarities and differences across studies in terms of the main analysis involving measured (correlational studies) or manipulated (experimental studies) hope used to draw conclusions about how hope relates to climate engagement. We defined an analysis as cross-sectional if all variables included were measured or manipulated in a single survey or a single timepoint. We defined an analysis as change-score if post-scores were subtracted from pre-scores and the resulting values were used in analyses. We defined an analysis as longitudinal or delayed engagement if outcome variables were measured at a different time than predictor variables (e.g., hours, days, or months later). We were flexible with categorization to accommodate different related choices of analyses; for example, we counted path models with indirect effects tests as mediation analyses.

Table 3 summarizes papers’ primary assessments of the relationships between hope and climate engagement and demonstrates that few studies examined how hope might sustain or motivate climate action over time. All correlational studies were cross-sectional (i.e., measured hope and engagement at only a single timepoint). Twenty-one of 24 experimental studies assessed climate engagement immediately after a manipulation, with only three examining engagement later in time to consider whether hope manipulations might exert sustained effects on engagement. Similarly, while many correlational (6 of 22) and experimental (12 of 24) studies conducted mediation analyses, the vast majority of these analyses were cross-sectional, meaning that causal time-order could be theoretically proposed but not demonstrated by the analysis.

**Table 3:** Designs of studies included in meta-analysis.

Study design	# studies
*Correlational studies*	
Zero-order correlation, cross-sectional design	2
Multiple regression controlling for covariates, cross-sectional design	13
Cross-sectional mediation, hope as mediator	6
Moderation analysis, cross-sectional design	1
Total correlational studies	22
*Experimental studies*	
Causal test of manipulation(s) on immediate engagement, no mediation	12
Causal test of manipulation(s) on immediate engagement + cross-sectional mediation w/hope	5
Cross-sectional mediation w/hope, no causal test of manipulation(s) on engagement	4
Causal test of manipulation(s) on immediate engagement + cross-sectional mediation w/other variable	1
Causal test of manipulation(s) on delayed engagement w/change scores, no mediation	1
Causal test of manipulation(s) on delayed engagement + change-score mediation w/hope	1
Causal test of manipulation(s) on delayed engagement + longitudinal mediation w/hope	1
Total experimental studies	24

For the meta-analysis itself, we were interested in direct relationships, so we focused solely on zero-order correlations and mean differences on engagement between conditions. Results including covariates (e.g., from multiple regressions, mediation analyses, and structural equation models) were not included in our meta-analyses. Thus, in some cases, statistics used in the meta-analysis could differ from the primary presentation of results in included papers (e.g., if conclusions in the primary paper were based on the results of a multiple regression or a mediation model).

### Overview of analyses

We used the metafor package (Viechtbauer, 2010) in R to conduct analyses. We used random effects models (as suggested by Borenstein et al., 2021). Because many studies contained multiple non-independent statistics (e.g., an experimental study with multiple types of hope manipulations, a study with multiple measures of hope or with multiple outcomes), all results below are based on three-level meta-analyses with nesting by non-orthogonal comparison (see Harrer et al., 2021) and maximum likelihood estimation (which allows comparison of nested models). Multiple studies within a paper are considered orthogonal, and in the experimental section, multiple experimental manipulations in a factorial design are considered orthogonal (Higgins et al., 2022). For simplicity, and because this information was often missing from studies, we used the program’s default assumption that results nested within a single orthogonal comparison unit were correlated at Pearson’s *r* = 0.50. Examining studies where this information was provided suggested that this was in many cases a reasonably accurate assumption.

To reduce confounds, when there were multiple comparison conditions, we used the condition that most closely related a climate-related neutral condition as the reference comparison. For example, when studies had both “fear appeal” and neutral (i.e., without an emotional appeal) comparison conditions, we used the neutral condition as the reference comparison. When studies had both non-climate-related and climate-related neutral conditions, we chose the climate-related neutral condition as the reference comparison.

To capture variance in effect sizes across studies, we report the Q-statistic and the 95% prediction interval (PI). If there are no systematic differences in effect size across studies, the Q-statistic should approximate the number of studies minus 1 (i.e., the number of degrees of freedom). Larger Q-statistics suggest greater variance. An accompanying value of *p* < 0.05 suggests that the variance across studies is unlikely to have occurred due to chance alone. The prediction interval provides an interpretable range for the extent of variance on the same metric as the effect sizes. The prediction interval has a very different interpretation from the confidence interval: the confidence interval quantifies the level of uncertainty in the mean estimate for the combined results across studies, while the prediction interval quantifies the dispersion of effect sizes across studies (Borenstein et al., 2021).

Below, we present the results in three sections. First, we meta-analyze the results from all studies combined (RQ1 & 2). Second, we examine the correlations between measures of hope and climate engagement and test whether results differ based on the target of measured hope (RQ3). Third, we examine the effects of experimental manipulations of hope and test whether results differ based on the type of experimental manipulation (RQ4) and explore the strength of experimental manipulation on hope assessed *via* differences across conditions in a post-manipulation measure of hope.

### Overall relationships

We conducted initial tests of the relationship between hope and climate engagement (RQ1) by converting all effect sizes from experimental and correlational studies to Fisher’s z. We back-transformed results to the metric of r for easier interpretation. A meta-analytic test with results nested by study demonstrated that, on average, hope was positively correlated with climate engagement, *r* = 0.13, *p* < 0.001, 95% CI [0.06, 0.20], with substantial heterogeneity in the effect sizes, *Q*(115) = 5,990, *p* < 0.001, 95% PI [−0.37, 0.57]. Estimates of variance components at different levels suggested variance at both level 3 and level 2, τ^2^_lvl3_ = 0.04, τ^2^_lvl2_ = 0.03, supporting using a three-level model. Moderation analysis demonstrated that effect sizes differed based on whether hope was manipulated [i.e., examining effects of experimental manipulations vs. measured (i.e., examining correlations)], *Q*(1) = 10.43, *p* = 0.001, with outcomes on average more strongly (positively) related to measured hope than manipulated hope (see below for details of each of these two categories).

#### Considering possible publication bias

**Figure 3:**
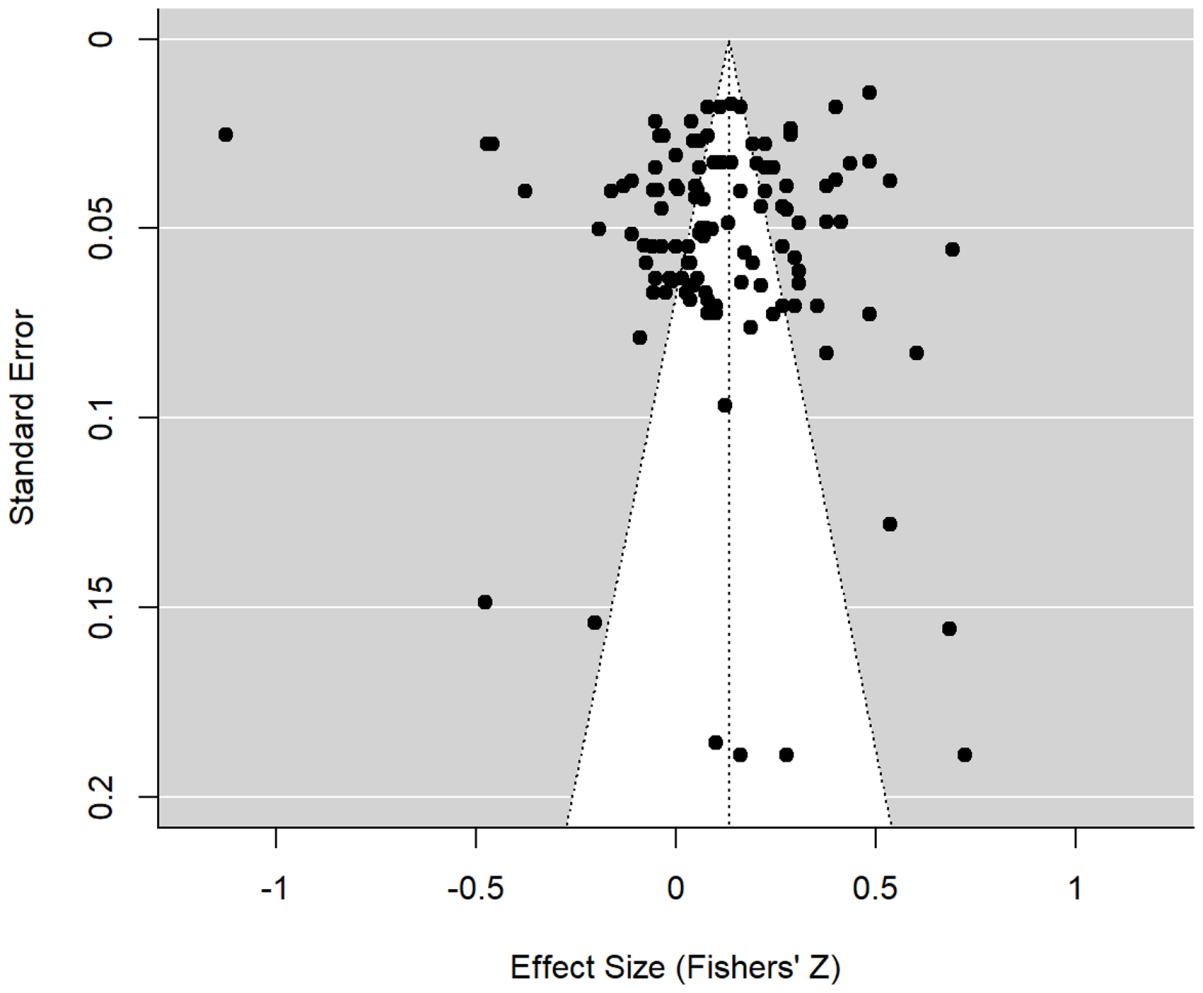
Funnel plot of effects by standard error.

Because we did not examine unpublished literature, we could be systematically underrepresenting work with null results because null results could be less likely to be published. This systemic bias against null results, if it exists, could lead to overestimating the true effect size. If null results are underrepresented in our work, we would expect that among the studies that did make it into the analyses, those with smaller (vs. larger) sample sizes should tend to produce larger effects (since large effects are needed to reach statistical significance with small sample sizes). We examined this possibility in two ways. First, we visually examined the funnel plot shown in Figure 3, which did not suggest that studies with greater standard errors (i.e., smaller sample sizes) systematically had greater effect sizes than those with smaller standard errors. Second, we conducted an Egger’s regression test. Broadly speaking, this test examines the relationship between study size and effect sizes; a significant result would typically suggest a systematic exclusion of small studies with null results, thus suggesting publication bias. The test was not significant, *z* = 1.71, *p* = 0.09, meaning that systematic exclusion was not identified. Taken together, this suggests that the impact of publication bias is probably trivial and that if other relevant but unpublished studies were included, we would not expect a systematic change in the estimated effect size (see Borenstein et al., 2021).

### Engagement outcomes (RQ2)

Studies examined a variety of engagement outcomes. We developed five types of climate change engagement based in part on previously work considering different types of pro-environmental outcomes (Stern, 2000; Geiger et al., 2019b). First, a few studies examined participants’ *desire to learn* about climate change (*k* = 3). Second, many studies examined *private-sphere* mitigation engagement (*k* = 16), such as taking public transportation or eating less meat. Third, many studies examined *public-sphere* mitigation engagement (*k* = 23), such as advocacy with government officials, collective action, and discussing climate change with others. Fourth, several studies examined *policy support* for climate change mitigation policy (*k* = 15). Fifth, a couple of studies examined climate *adaptation* engagement (*k* = 2), such as farmers adapting to drier conditions.^3^ Some studies included multiple measures falling into different categories.

A second moderator considered how engagement was assessed using common distinctions between actual measured behaviors and proxies for these behaviors (e.g., Maki et al., 2019). We divided studies into four categories: actual *measured* behavior (*k* = 5), *self-report* assessments of behavior (*k* = 15), behavioral *intentions* (*k* = 16), and *abstract* measures of engagement (*k* = 20), such as willingness to engage in a behavior or to pay for renewable energy. As before, some studies included multiple measures that fell into different categories.

Across all studies, findings were not moderated by type of climate engagement, *Q*(4) = 3.71, *p* = 0.45 nor assessment of engagement, *Q*(3) = 0.80, *p* = 0.85.

### Measures of hope and climate engagement (RQ3)

We next examine associations between measures of hope and climate engagement. We first present an overview of our coding. Then we present meta-analyses on all studies that measured hope and moderation analyses by subgroup.

#### Overview of hope measures

##### Target of hope

Following an extensive examination of the conceptualization and operationalization of hope measurements in the articles, and weighting *a priori* considerations alluded to in the introduction, we identified six categories of targets of hope. First, a few studies (*k* = 3) examined *domain-general* hope (i.e., asking about how hopeful participants generally were without mentioning climate change). Second, several studies (*k* = 9) measured hope about *climate change* generally. Based on the vagueness of these measures, participants may vary in aspects of climate change they may have in mind, and different studies might yield systematically different relationships by priming certain ideas before assessing hope with this type of measure. Third, several studies (*k* = 13) examined how hopeful participants were when contemplating a *goal* or solution to address climate change (we included measures of hope about scientific and political solutions that did not fit into the *actions* category). Fourth, several studies (*k* = 7) examined how hopeful participants were when contemplating *actions* they could personally take or participate in with others to address climate change. Fifth, several studies (*k* = 9) provided participants with a text or video message related to climate change (e.g., as an experimental manipulation) and subsequently asked participants how hopeful the *message* made them. Finally, some studies (*k* = 5) examined hope grounded in *denial* of the severity of climate change or the need to make major societal changes to address it (i.e., based on the possibility that climate change is not a major concern or would be solved by nonhuman forces). Some multiple-item measures of hope involved different items that could be placed into different categories; we categorized these measures based on which category a majority of items fell into. Some studies included multiple measures of hope that fell into different categories; these were included separately with non-independent correlations nested within studies.

##### Hope-as-feeling vs. hope-as-cognitive-state

We categorized hope measures in which survey items used the word “feel” as *feeling*. We categorized hope measures that assessed a cognitive state (e.g., Snyder’s Hope scale, Snyder, 2002; the “climate change hope scale,” Li and Monroe, 2018) as *cognitive state*. We categorized measures that did not meet either of these two criteria (e.g., “How hopeful are you”) as *neither*. In total, 23 studies measured hope as a feeling, 12 measured it as a way of thinking, and three studies’ measures did neither. One study (Pleeging et al., 2021) used multiple measures of hope and included “feeling” hope and “thinking” hope.

#### Meta-analytic results

**Figure 4:**
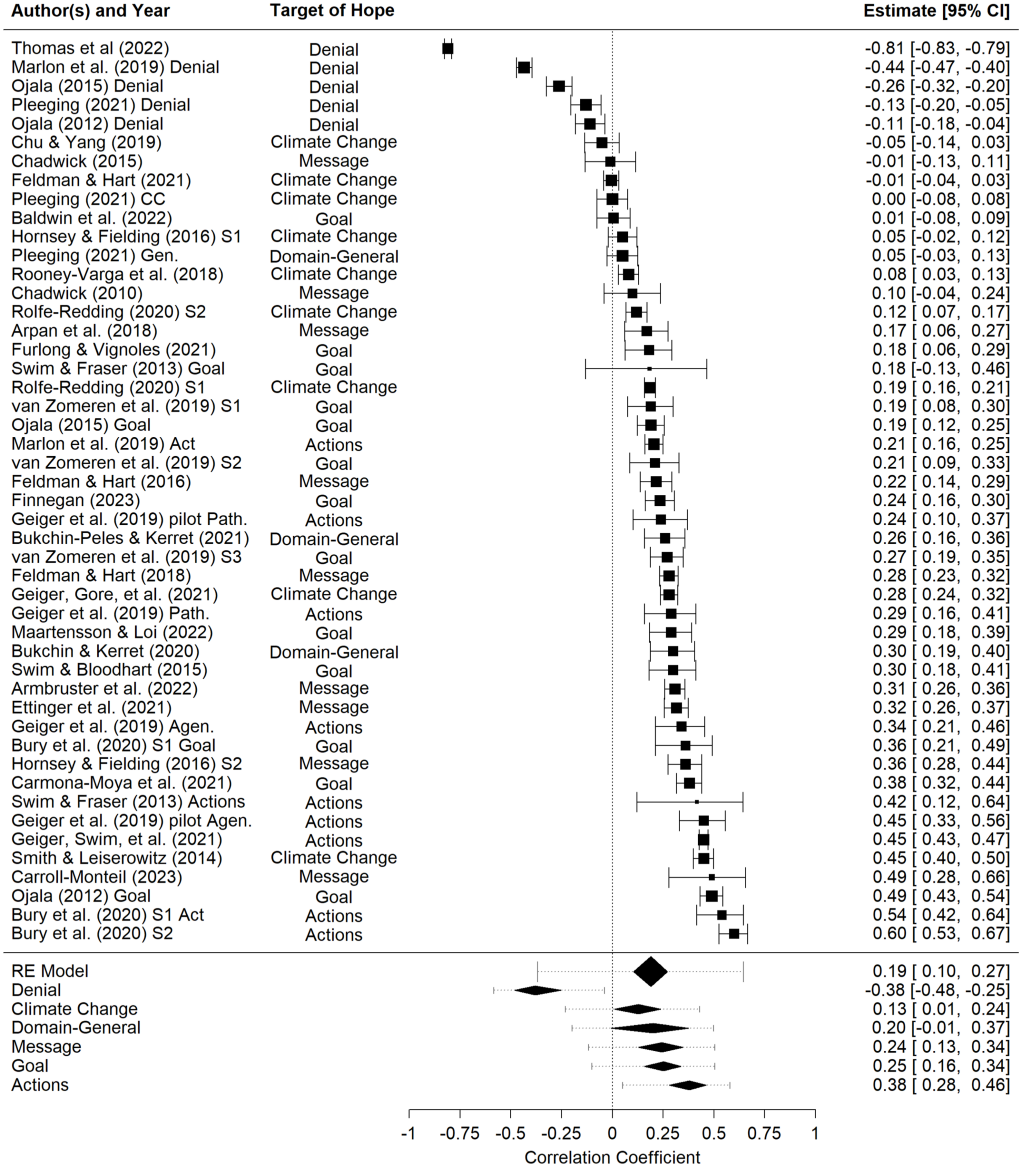
Forest plot of correlations between measured hope and climate engagement. For results by study, square sizes represent weights and solid lines represent confidence intervals. For summary statistics, diamonds represent confidence intervals and dashed lines represent prediction intervals.

We conducted a meta-analysis on correlational results only, with correlations converted to Fisher’s z. When presenting results, we back-transformed results to the metric of *r* for easier interpretation. On average, measured hope was associated with greater climate engagement, *r* = 0.18, *p* < 0.001, 95% CI [0.10, 0.26] with significant heterogeneity, *Q*(69) = 5,639, *p* < 0.001, 95% PI [−36, 0.63] (see Figure 4).

A moderator test showed that the target of hope moderated the relationship, *Q*(5) = 104, *p* < 0.001. As shown in greater detail in Table 4 and Figure 4, denial hope correlated negatively with climate engagement (*r* = −0.40), and this correlation was smaller than those related to all other targets of hope, *p*s < 0.05. In contrast, all other targets of hope yielded positive correlations with climate engagement (though domain-general was only marginally significant). Finally, hope elicited in response to contemplating action was particularly strongly correlated with engagement (*r* = 0.40) and more strongly correlated with climate engagement than hope about climate change more generally (*r* = 0.13), *p* < 0.05. There was unexplained between-study variance even after including this moderator, *Q*(64) = 1940, *p* < 0.001, indicating that there are other undiscovered moderators influencing results.

**Table 4:** Potential moderators of measured hope and climate change engagement.

Measure	Correlation coefficient	95% CI	95% PI
*Target of hope* [*Q*(*5*) *= 104, p < 0.001*]			
Denial (k = 5)	−0.40^***, a^	−0.52, −0.26	−0.67, −0.04
Climate change (k = 9)	0.13^*,b^	0.01, 0.24	−0.24, 0.46
Domain-general (k = 3)	0.20^+,b,c^	−0.01, 0.39	−0.20, 0.55
Message (k = 9)	0.25^***,b,c^	0.13, 0.36	−0.12, 0.55
Goal (k = 13)	0.26^***,b,c^	0.16, 0.35	−0.10, 0.56
Actions (k = 7)	0.40^***,c^	0.28, 0.50	0.05, 0.66
*Aspect of hope* [*Q*(*2*) *= 1.52, p = 0.47*]			
Cognitive state (k = 13)	14^*,a^	0.01, 0.27	−0.39, 0.61
Feeling (k = 25)	18^***,a^	0.08, 0.27	−0.35, 0.62
Neither (k = 3)	0.32^*,a^	0.07, 0.54	−0.26, 0.73
*Type of engagement* [*Q*(*4*) *= 1.99, p = 0.73*]			
Information-seeking (k = 1)	−0.05^a^	−0.49, 0.41	−0.65, 0.59
Policy support (k = 13)	0.13^+, a^	−0.004, 0.26	−0.40, 0.60
Public-sphere (k = 21)	0.19^***,a^	0.08, 0.29	−0.35, 0.63
Private-sphere (k = 13)	0.21^**,a^	0.07, 0.34	−0.33, 0.65
Adaptation (k = 2)	0.28^a^	−0.10, 0.59	−0.36, 0.74
*Assessment of engagement* [*Q*(*3*) *= 0.17, p = 0.98*]			
Intentions (k = 15)	0.16^*,a^	0.03, 0.28	−0.38, 0.62
Abstract (k = 18)	0.18^**,a^	0.06, 0.29	−0.36, 0.63
Measured (k = 2)	0.19^a^	−0.19, 0.52	−0.44, 0.69
Self-report (k = 12)	0.20^**,a^	0.07, 0.32	−0.34, 0.64
Average (k = 39)	0.18^***^	0.10, 0.25	−0.35, 0.63

Stars and the plus sign represent whether the overall effect of the type of manipulation is significantly different from zero: ^+^*p* < 0.10, ^*^p < 0.05, ^**^p < 0.01, ^***^p < 0.001. Within each moderator, values are significantly different from one another if they do not contain the same letter superscript. CI, confidence interval; PI, prediction interval. k = number of studies.

In contrast, the aspect of hope assessed (feeling vs. cognitive-state vs. neither) did not significantly moderate effects, *Q*(2) = 1.52, *p* = 0.47. Similar to the meta-analysis results above, examining only correlations between measured hope and engagement again showed that the relationships were not moderated by either type of engagement, *Q*(4) = 1.99, *p* = 0.73, nor assessment of engagement, *Q*(3) = 0.17, *p* = 0.98. See Table 4 for more details and Supplementary Table S1 for exploratory interactions between variables.

### Manipulations of hope and climate engagement (RQ4)

We next examine studies that manipulated hope and examined effects on climate engagement. We first present an overview of our coding. Then we present meta-analyses on all studies that manipulated hope and moderation analyses by subgroup.

#### Overview of manipulations

Following an extensive examination of the conceptualization and operationalization of the 33 hope manipulations in the articles and weighting *a priori* considerations alluded to in the introduction, we identified six categories of manipulations of hope. First, a few manipulations were classified as *status quo frames* (*k* = 3). These manipulations described the possibility of environmental conditions remaining the same in the future if action on climate change is taken (i.e., the potential for preservation of the status quo). Second, the most common method of manipulating hope was to convey *societal efficacy* (*k* = 12). These messages were designed to increase the sense of perceived possibility that society could address climate change; for example, focusing on recent policy changes expected to help address climate change, providing possible solutions to climate change (e.g., solar panels), or images of climate marches. These manipulations were intended to provide a sense of possibility that climate change could be addressed, and in some cases, specific actors and institutions who could assist in this transition but did not directly imply the possibility of the individual receiving the message participating in the solutions. Third, a couple of manipulations employed messages promoting *personal efficacy* (*k* = 2), for example, providing information that a government agency is likely to take feedback from average citizens into account when considering the extent of action to take on climate change. Fourth, some manipulations employed messages attempting to increase hope by increasing the perceived *importance* of addressing climate change (*k* = 6; some argue that importance is a key component of hope, e.g., Chadwick, 2015). Fifth, several manipulations *combined* two or more of these components into one message (*k* = 8). Finally, a couple of studies employed a sixth type of manipulation that did not involve messaging and instead involved participating in an *in-depth* experience such as a training program or computer simulation (*k* = 2).

#### Meta-analytic results

We conducted a meta-analysis on effects of experimental manipulations on climate engagement, with effect sizes in Cohen’s D (i.e., standardized mean difference between conditions). On average, there was a small but statistically significant effect of the hope manipulations on climate engagement, *d* = 0.08, *p* = 0.04, 95% CI [0.002, 0.17] with substantial heterogeneity, *Q*(45) = 202, 95% PI [−0.31, 0.48].

A subgroup analysis revealed that effects differed by type of manipulation, *Q*(5) = 28.6, *p* < 0.001. As shown in Table 5, results suggested that the *status quo frame* messages marginally decreased climate engagement, *d* = −0.19, *p* = 0.06, and were significantly worse at increasing climate engagement than other manipulations, *p*s < 0.05. In contrast, the *personal efficacy* manipulations (*d* = 0.18, *p* = 0.07) and *importance* manipulations (*d* = 0.12, *p* = 0.09) marginally increased climate engagement. Further, the *in-depth* interventions increased climate engagement, *d* = 0.49, *p* < 0.001, and were significantly better at increasing climate engagement than all other categories of interventions. Neither *societal efficacy* nor *combined* interventions significantly impacted climate engagement, *p*s > 0.10. After the moderator of type of manipulation was included, there was still significant heterogeneity, *Q*(40) = 103, *p* < 0.001, suggesting the possibility of at least one undiscovered moderator. Similar to the meta-analysis results above, examining only effects of manipulations on engagement again showed that the effects of manipulations did not differ based on type of engagement, *Q*(3) = 0.44, *p* = 0.93 nor assessment of engagement, *Q*(3) = 6.35, *p* = 0.10. See Table 5 for more details and Supplementary Table S2 for exploratory interactions between variables.

**Table 5:** Potential moderators of manipulated hope and climate change engagement.

Moderator	SMD (SE)	95% CI	95% PI
*Type of manipulation* [*Q*(*5*) *= 28.6, p < 0.001*]			
Status quo frame (k = 3)	−0.19 (0.10)^+,a^	−0.39, 0.01	−0.50, 0.11
Combined (k = 8)	0.04 (0.06)^b^	−0.08, 0.16	−0.22, 0.30
Societal efficacy (k = 12)	0.05 (0.04)^b^	−0.04, 0.14	−0.20, 0.29
Importance (k = 6)	0.12 (0.07)^+,b^	−0.02, 0.25	−0.15, 0.38
Personal efficacy (k = 2)	0.18 (0.10)^+,b^	−0.02, 0.39	−0.12, 0.49
In-depth (k = 2)	0.49 (0.10)^***,c^	0.31, 0.68	0.20, 0.79
*Type of engagement* [*Q*(*3*) *= 0.44, p = 0.93*]			
Information-seeking (k = 2)	0.04 (0.09)^a^	−0.13, 0.21	−0.38, 0.46
Public-sphere (k = 13)	0.08 (0.06)^a^	−0.03, 0.19	−0.32, 0.48
Policy support (k = 11)	0.09 (0.05)^a^	−0.02, 0.19	−0.31, 0.51
Private-sphere (k = 13)	0.09 (0.06)^a^	−0.03, 0.20	−0.31, 0.49
*Assessment of engagement* [*Q*(*3*) *= 6.35, p = 0.10*]			
Intentions (k = 16)	0.05 (0.05)^a^	−0.05, 0.14	−0.31, 0.40
Abstract (k = 14)	0.05 (0.05)^a,b^	−0.05, 0.15	−0.31, 0.41
Measured (k = 7)	0.19 (0.09)^*,a,b^	0.02, 0.37	−0.20, 0.58
Self-report (k = 2)	0.33 (0.14)^*,b^	0.06, 0.60	−0.11, 0.77
Average/Total (k = 33)	0.08 (0.04)^*^	0.002, 0.17	−0.31, 0.48

Stars and the plus sign represent whether the overall effect of the type of manipulation is significantly different from zero: ^+^*p* < 0.10, ^*^p < 0.05, ^***^p < 0.001. Within each moderator, values are significantly different from one another if they do not contain the same letter superscript. SMD, standardized mean difference between conditions (i.e., Cohen’s D). SE, standard error; CI, confidence interval; PI, prediction interval. k = number of manipulations.

#### Does the effectiveness of manipulations on engagement differ based on increases in hope?

We were able to obtain effect sizes for the degree to which 26 (out of 33) hope manipulations (vs. control conditions) increased hope. The average increase in hope from manipulations was only small-to-moderate in size, *d* = 0.31, *SE* = 0.06, *p* < 0.001, 95% CI [0.20, 0.43]. Multiplying this average increase in hope by the average correlation between hope and climate engagement noted above (*r* = 0.18) yields a point estimate of the indirect effect of the manipulations through hope of *d* = 0.06,^4^ which is fairly similar to the point estimate for the actual effect of manipulations (*d* = 0.08).

There was substantial variance in the extent to which different manipulations increased hope, *Q*(35) = 232, *p* < 0.001; 95% PI [−0.21, 0.83]. However, increases were similar regardless of whether participants were asked how hopeful the message they received made them or were asked about other targets of hope (e.g., climate change), *Q*(1) = 0.32, *p* = 0.57. Similarly, different types of manipulations had statistically similar effects on hope, *Q*(5) = 8.45, *p* = 0.13 (see Supplementary Figure S1 for details).

**Figure 5:**
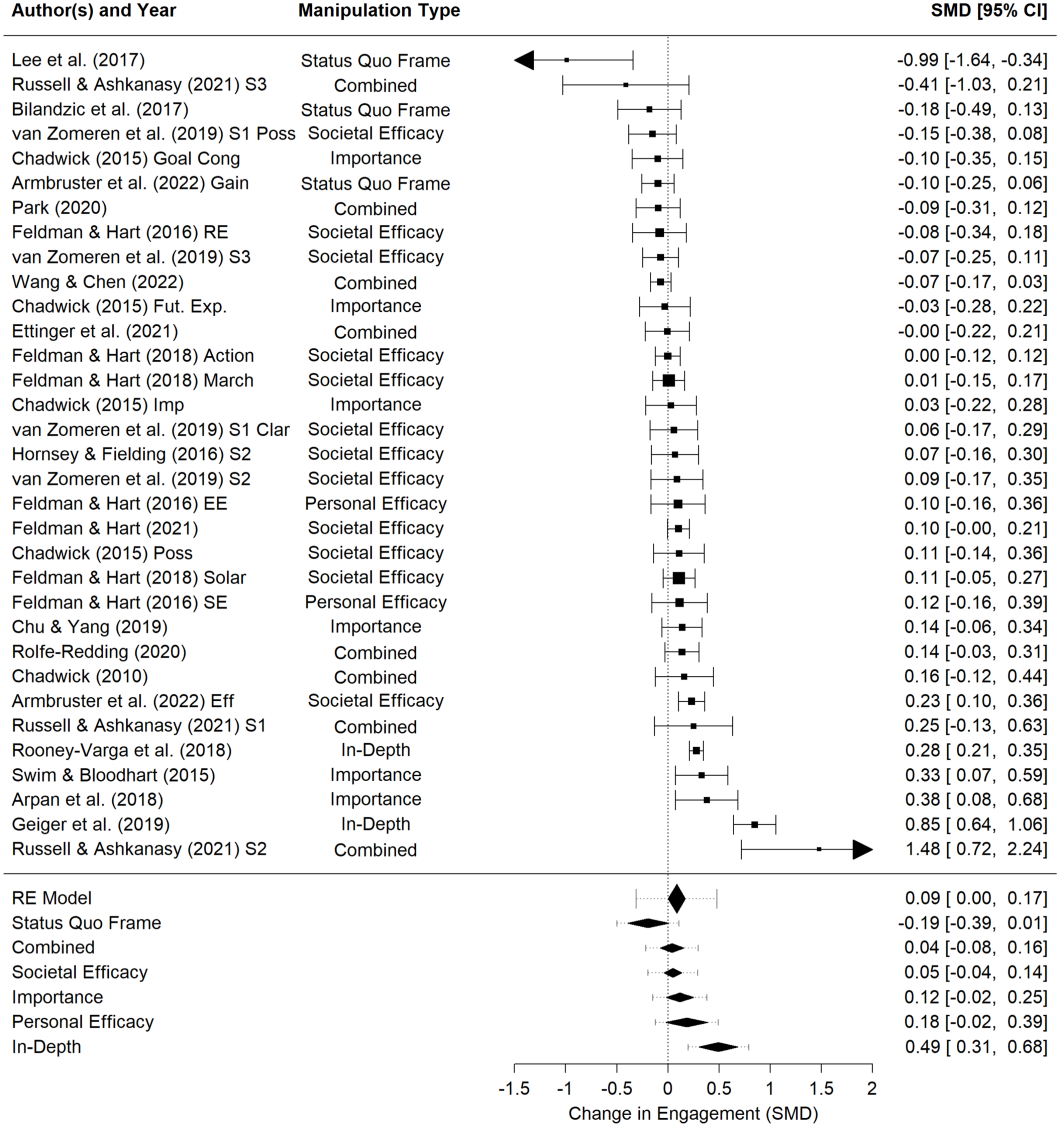
Forest plot of manipulation effectiveness at increasing climate engagement. For results by study, square sizes represent weights and solid lines represent confidence intervals. For summary statistics, diamonds represent confidence intervals and dashed lines represent prediction intervals. SMD, Standardized Mean Difference between conditions (i.e., Cohen’s D).

**Figure 6:**
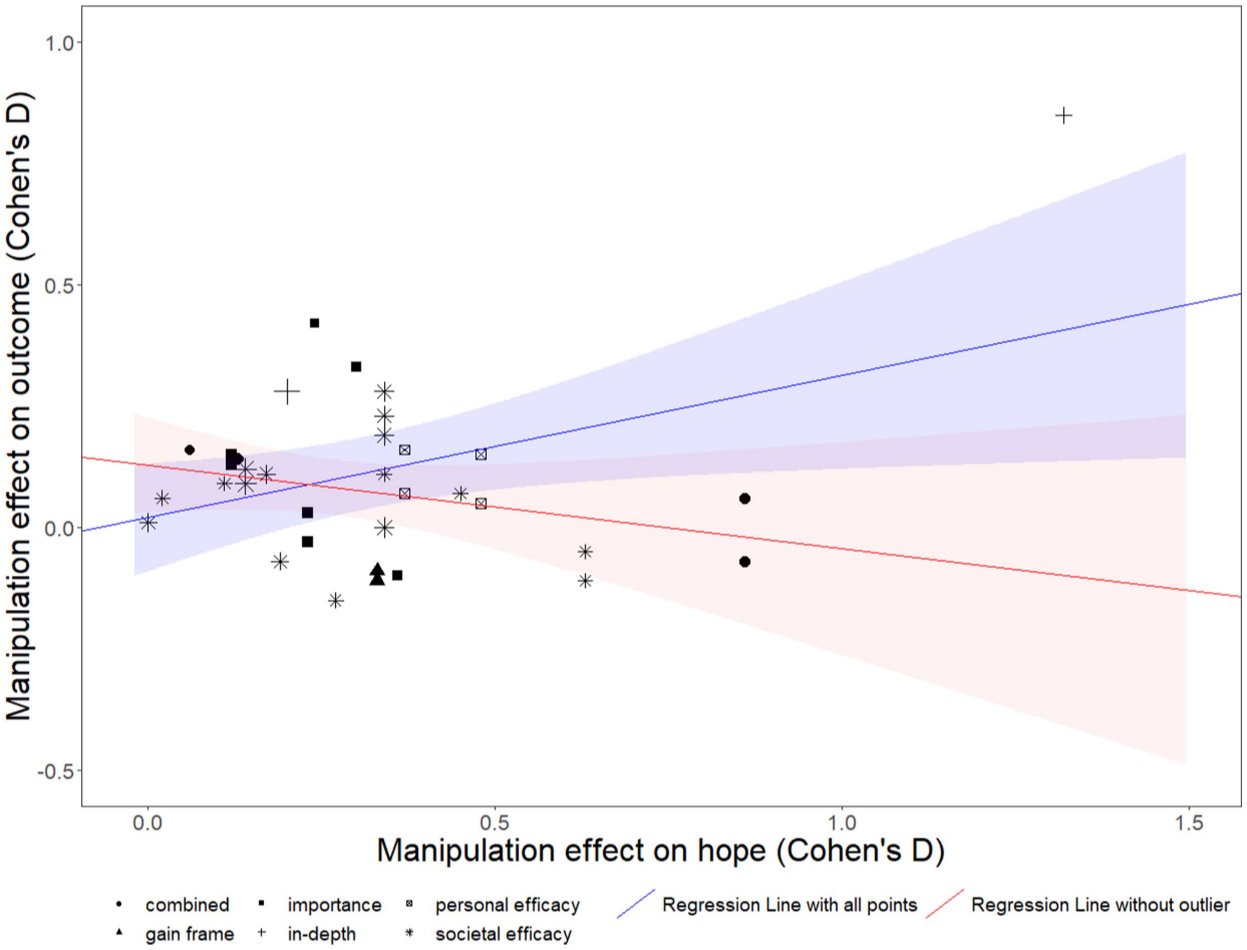
Between-study relationship between manipulation effectiveness on increasing hope and effectiveness at increasing engagement. Shaded areas represent 95% confidence intervals.

We assessed whether manipulations that yielded a greater increase in hope also yielded a greater increase in climate engagement. An initial look at this relationship suggested that manipulations that yielded greater increases in hope also yielded greater increases in climate engagement, *b* = 0.30, *SE* = 0.13, *p* = 0.02, 95% CI [0.05, 0.54] (see the blue line in Figure 5). However, visually examining the scatterplot revealed a single high-leverage outlier (see the top right of Figure 6); corresponding to a study from Geiger et al. (2019a). Excluding this outlier, the effect became nonsignificant and sloped in the opposite direction, *b* = −0.14, *SE* = 0.16, *p* = 0.40, 95% CI [−0.46, 0.17] (see the red line in Figure 6). Thus, excluding this outlier, there is no systematic relationship between the effectiveness of the manipulations at increasing hope and how effective they were at promoting climate engagement.

## Discussion

### Are hopeful people engaged with climate change?

On average, reporting more hope was associated with greater engagement with climate change. This positive association did not significantly differ based on type of engagement outcome (e.g., policy support vs. behavior) or how the outcome was assessed (e.g., behavioral intentions vs. measured behavior). Similarly, correlations between hope and engagement did not significantly differ whether hope was measured as a feeling versus a cognitive state.

In contrast, we found that the target of people’s hope is important when considering whether hope leads to engagement. Consistent with the argument made by Geiger et al. (2021b), we found that hope in response to contemplating taking action on climate change was the most strongly associated with climate engagement (*r* = 0.40), and was significantly stronger than hope elicited in response to climate change more generally (*r* = 0.13). Hope elicited in response to considering societal action toward climate change and hope when reflecting on climate change-related messages were positively associated with climate engagement (*r*s = 0.25, 0.26) and the average correlation for each of these two categories was in between the two above correlations (though not significantly different from either of the above). Conversely, and consistent with Ojala (2012a), those reporting greater hope based on the perception that climate change is not a problem or would not require intervention (i.e., *denial* hope) tended to be less likely to engage with climate change. Taken together, these findings suggest that to understand the extent to which hope is associated with climate action, one must consider what people feel hopeful about.

### Hope manipulations may only slightly increase immediate climate engagement

Our meta-analysis of experimental studies lends partial credence to and qualifies observation of Ojala (2022) that most hope appeals tested in published work do not appear to impact climate change engagement. Our meta-analytic results demonstrate a significant but small (*d* = 0.08) and heterogeneous effect of hope manipulations on climate engagement.

Subgroup analysis by type of manipulation in the present meta-analysis suggests the promise of two types of manipulations with few tests: *in-depth* and *personal efficacy* manipulations. In-depth manipulations (*k* = 2), which were time-consuming experiences designed to boost hope and climate engagement, were the most effective type of manipulation and significantly increased engagement (*d* = 0.49). Similarly, *personal efficacy* manipulations (*k* = 2), which were messages explaining how the message recipient could personally contribute to climate action, exerted a marginally significant positive impact on increasing engagement (*d* = 0.18). Despite these promising preliminary trends, the small number of tests in each of these categories, and *personal efficacy* effects being only marginally significant, demonstrates the need for further tests of these types of manipulations on climate engagement (Figure 6).

Hope manipulations that focused on increasing *societal efficacy* (e.g., providing a sense of possibility that climate change could be solved) were the most common type of manipulation (*k* = 12) and thus arguably reflect the most common conceptualization of a “hopeful message.” Therefore, it is informative that they did not significantly or substantially increase climate engagement (nor decrease climate engagement; *d* = 0.05). It is possible that these manipulations could be more effective at increasing engagement under the right boundary conditions, such as with populations already highly motivated to engage or who already have a sense of personal efficacy (e.g., see Cohen-Chen and Van Zomeren, 2018). Alternatively, these manipulations might not immediately promote climate engagement but could sustain engagement over time (Ojala, 2016). Nonetheless, the fact that our meta-analysis did not demonstrate an effect of manipulations should give advocates of these messages pause and suggests the need for theoretically-informed study designs differing from those used here to empirically test whether these manipulations can be more effective at promoting climate engagement than our meta-analysis suggests.

Finally, *status quo frames* marginally decreased climate engagement and were significantly less effective than other types of hope manipulations. A possible confound with this finding is that these studies’ design did not allow for a proper control condition, so we compared these manipulations to loss frames that highlighted severe negative impacts if climate change were not addressed. Thus, these studies might instead demonstrate the motivational power of loss-framed messages (i.e., highlighting severe negative impacts resulting from inaction on climate change) rather than the demotivational power of the status quo-framed messages. It is also worth noting that the authors termed these manipulations gain frames, which we believe is technically accurate but does not fit how we would conceptualize a gain frame. These studies used *equivalence framing* (Scheufele and Iyengar, 2017), which seeks to present identical information through different lenses (e.g., outcomes if action is not taken in the “loss frame” vs. outcomes if action is taken in the “gain frame”). This type of study design has been argued to be superior at avoiding possible confounds (see Scheufele and Iyengar, 2017), but as a result, these studies did *not* communicating gains or co-benefits from addressing climate change (e.g., social justice; public health, economic benefits; see Myers et al., 2012; Bain et al., 2016; Bergquist et al., 2020; Geiger et al., 2021b) which is how we would conceptualize a gain frame in this context. Instead, they focus on the possible maintenance of the status quo. Future research should consider whether messages focusing on true gains motivate action more effectively than those focusing on the possibility of preserving the status quo.

#### Are small increases in engagement due to relatively weak manipulations?

It is worth considering whether the small effect sizes of experimental manipulations could be explained by their relatively small effectiveness at increasing hope. On average, experimental manipulations yielded a small-to-medium increase (*d* = 0.31, see Cohen, 1988) on measures of hope assessed after the manipulation. This relatively small effect size does not appear unusual for effects on a distal outcome (in this case, climate engagement), but we would generally expect that that experimental manipulations should exert medium-to-large effects on proximal outcomes and manipulation checks (in this case, measured hope).^5^ Perhaps it is difficult to increase substantially and reliably increase hope about climate change-related topics through short messages (e.g., see Hornsey et al., 2021). This suggests that perhaps the relatively weak effect of manipulations on promoting engagement might be due to their relatively weak effect on increasing hope. Indeed, the point estimate for the indirect effect of manipulations through increased hope (*d* = 0.06), based on the average correlation between hope and engagement, is similar to the actual point estimate for the effect of experimental manipulations (*d* = 0.08). This suggests that experimental manipulations of hope might be increasing climate engagement about as much as expected given their strength at increasing hope and the correlational results between hope and engagement.

Yet, other analyses provide a cautionary note against the assumption that strengthening hope manipulations’ effectiveness at increasing hope would also yield stronger increases in climate engagement. If this were the case, one would expect that across studies in our meta-analysis, those that were more effective at increasing hope would also be more effective at increasing climate engagement. It was true that this pattern was initially confirmed, yet, after excluding one high-leverage outlier that greatly increased both hope and climate engagement, the relationship became nonsignificant and switched directions. If anything, these non-significant results suggested that the most effective manipulations might be increasing hope less than the less effective ones.

Overall, these patterns do not conclusively answer whether creating manipulations that are better at increasing hope would foster larger increases in climate engagement. The relatively small number of experimental studies and the multitude of possible confounds across studies leave open this possibility, despite our inability to detect it. However, it is also worth considering the contrasting possibility that increases in hope may be incidental to the effectiveness of these manipulations. That is, it is possible that manipulations are increasing climate engagement due to some other process unrelated to hope, and also separately increasing hope. For example, it is possible that manipulations are increasing efficacy and the increases in efficacy are independently increasing both engagement and hope (e.g., see Van Zomeren et al., 2019). In this case, increased hope would not be a causal prerequisite for climate engagement but rather an incidental side effect of other processes leading to engagement. Future work is needed to tease apart these constructs and more fully explore this speculation.

### Limitations and future directions

Conclusions from our meta-analysis are limited by the existing body of published research that we located. Our review demonstrates that most studies on this topic recruited student or general population samples; few recruited groups already engaged with climate change (e.g., activists; farmers). Our review also highlights that most published work on this topic has sampled participants from the Global North (i.e., wealthier countries with higher education levels, who tend to be relatively less impacted by climate change), and especially the United States, a country which in some ways is a global outlier regarding climate change views (Geiger et al., 2022a), which reflects field-wide systematic biases (Comfort and Park, 2018; Tam et al., 2021). Additionally, despite our systematic screening and selection process, given our search’s interdisciplinary nature, we likely missed some relevant articles. Finally, our review is specific to engagement with climate change. Future work should examine how patterns might differ for other environmental and non-environmental issues. For example, Lee et al. (2017) provide preliminary evidence that status quo frames (or gain frames) might be more motivating for local (vs. global) environmental issues. It is possible that the preference to maintain the status quo is more motivational for concrete issues (e.g., local environmental issues) vs. abstract issues (e.g., global environmental issues; Yudkin et al., 2019).

Future work is needed to more fully explore questions in which limited information was available to include in the meta-analysis. For example, some manipulations had only a small number of studies included (e.g., *personal efficacy* manipulations; *in-depth manipulations*); future work should provide additional tests of the effects of these types of manipulations on climate engagement. Additionally, few studies to date have examined the relationship between domain-general hope and climate engagement. This relationship might differ based on how domain-general hope is assessed. The studies here used Snyder’s trait hope index which other work has shown correlates with pro-social behavior (see Schornick et al., 2023 for a systematic review), however, it is possible that other measures of domain-general hope might better align more strongly with climate engagement. Finally, future research should explore the effects of highlighting possibility of climate action yielding co-benefits (e.g., job creation, social justice, and positive health implications of the energy transition) rather than the potential to preserve the status quo. Finally, work is needed to explore other outcomes that were not identified through present literature search, including cooperative behavior, trust in leaders who promote action on climate change, and creative work toward solutions (e.g., see McAfee et al., 2019; Geiger et al., 2022b). It is possible that hope might have unique impacts on these and other outcomes that were not included here.

Future work is needed to systematically examine whether including other message components or interventions in combination with hope manipulations increases the hope manipulations’ effectiveness. For example, one study included in our meta-analysis Armbruster et al. (2022) found that a message that combined an efficacy component (which induced more hope than omitting this component) with a loss frame (which induced less hope than a gain or non-loss frame) frame was more effective than any other combination despite inducing less hope than the efficacy and gain frame combination. The effectiveness of this combination is consistent with the extended parallel process model (Witte, 1992), which suggests that a combination of risk and efficacy is likely to be most motivating (also see Tannenbaum et al., 2015). Another example demonstrated in other domains of collective action (Cohen-Chen and Van Zomeren, 2018) showed that collective action on the topics was most likely when messages include both a hope appeal and a group efficacy-boosting component.

Our meta-analysis did not conclusively demonstrate that increasing hope increases climate engagement. The positive correlations with measured hope could potentially be largely explained by either engagement fostering hope (e.g., Ojala, 2022), or an unmeasured third variable affecting both hope and engagement. Preliminary but weak evidence against reverse causation (i.e., climate engagement fostering hope) is provided in the Supplementary material; future work is needed to test this question adequately. We alluded to the “third variable” possibility above when discussing the lack of relationship between increased hope and increased engagement in experimental studies. Additional evidence for this possibility comes from Van Zomeren et al. (2019). They demonstrate that positive correlations between hope and climate engagement disappear or even reverse when controlling for efficacy. This suggests the possibility that efficacy could both promote climate engagement, and separately, foster hope.

Researchers should also consider using longitudinal designs to better align with theoretical perspectives on how emotions indirectly motivate action over time. For example, Baumeister et al. (2007) argue that emotions most typically play an indirect role in behavior by shaping cognitive processing of experiences over time (also see Chapman et al., 2017). Similarly, Ojala (2012b, 2016) argues that hope is implicated in meaning-focused coping, whereby it acts as a buffer facilitating confrontation of sources of negative feelings and sustained engagement with difficult issues over time. Supporting this explanation, it is worth noting that both in-depth manipulations (the category of manipulation that had by far the largest impact) occurred over a longer timespan than other studies and assessed changes in engagement over time rather than immediately after receiving a message. Yet, in total, only three studies eligible for our meta-analysis (Rooney-Varga et al., 2018; Geiger et al., 2019a; Wang and Chen, 2022) collected data at more than one timepoint and even these studies did not collect the appropriate data to fully explore this hypothesis. Similarly, most studies that included mediation analyses related on cross-sectional data, meaning that they could not provide causal evidence for processes proposed to unfold over time. Future work should consider using study designs that allow for the use of longitudinal mediational analyses. Table 6 summarizes limitations and recommendations for future research.

**Table 6:** Limitations and possibilities for future research.

Limitation	Possible future research	References of interest
Most research studied convenience samples in Global North	Study more diverse populations. Study more specific groups of interest (e.g., activists; businesspeople; and politicians)	Bukchin and Kerret (2020); Bukchin-Peles and Kerret (2021); Comfort and Park (2018); Furlong and Vignoles (2021); Geiger et al. (2019a)
Few tests of manipulations with strongest impacts	Test effects of messages that appeal to hope through suggesting personal efficacy on climate engagement. Test effects of hope-boosting experiential learning on engagement. Explore psychological mechanisms boosting engagement	Feldman and Hart (2016); Geiger et al. (2019a); Rooney-Varga et al. (2018)
Our exploration of boundary conditions was limited	Consider exploring whether hope manipulations are most effective when other components are also included. Consider whether effects of hope might vary based on individual differences	Armbruster et al. (2022); Cohen-Chen and Van Zomeren (2018); Feldman and Hart (2016)
Hope about climate change generally only weakly associates with climate engagement	Consider increasing specificity of questions assessing hope (e.g., asking about specific targets of hope)	Geiger et al. (2021c); Swim et al. (2023)
Few tests of relationship between domain-general hope and climate engagement	Examine whether domain-general hope is associated with climate engagement. Consider measures of domain-general hope shown to associate with pro-social behavior	Bukchin and Kerret (2020); Bukchin-Peles and Kerret (2021); Pleeging et al. (2021)
Lack of conclusive evidence of motivating effect of hope	Demonstrate that hope predicts behavior above and beyond efficacy. Show that hope is uniquely important to the success of a manipulation. Consider longitudinal analyses (see below)	Cohen-Chen and Van Zomeren (2018); Skurka et al. (2023); Van Zomeren et al. (2019)
Little research with measured behavior measured ecologically valid behaviors for addressing climate change	Examine cooperative outcomes (e.g., trust, willingness to cooperate with others, actual cooperation; provision of emotional support to others)	Balliet et al. (2011); Parks et al. (2013); Swim et al. (2018); van Swol et al. (2022)
Most research used single-timepoint designs	Consider measuring hope and engagement at multiple timepoints after an intervention. Consider effects of repeated exposure to a message. Use longitudinal analyses	Geiger et al. (2019a); Skurka et al. (2023)

## Conclusion

Our findings provide essential insights into whether and when hope might be most likely to motivate climate engagement. A meta-analysis of correlational studies demonstrated that hope was, on average, associated with greater climate engagement but this association differs based on the target of hope. In particular, hope related to solutions, and even more so, hope about specific actions, are robustly associated with greater climate engagement. In contrast, hope about climate change in general is positively but weakly correlated with climate engagement. Conversely, hope grounded in denial of climate change is associated with less engagement. Similarly, although evidence is sparser with the experimental studies, hopeful messages promoting the efficacy of the respondent taking action (i.e., *personal efficacy*) and *in-depth* experiences where participants engaged in experiential learning showed promise as possibly effective types of hope manipulations that could boost climate engagement. In contrast, hopeful messages promoting a sense of possibility that society could address climate change neither substantially promoted nor discouraged engagement. Researchers should consider different study designs (e.g., longitudinal designs) appropriate to capture change aligned with theoretical perspectives on how hope promotes climate engagement. Although future work is needed to yield greater confidence in these findings, our meta-analysis provides preliminary evidence that hope about climate change—construed broadly—may not be as impactful at promoting engagement as hope that one can personally take action on the issue to create a better future.

## Data availability statement

Analyses were conducted based on previously existing datasets and information which was publicly available. This data can be found at: https://osf.io/3wku4.

## Author contributions

NG: study conceptualization, manuscript drafting, supervision, examination of articles included in review, and data analysis. TD: lead examination of abstract and full text of all articles, manuscript edits, and critical feedback JS: manuscript edits, examination of articles included in review and feedback on analysis. All authors contributed to the article and approved the submitted version.

## Funding

This research was partially supported by NSF Grant 2129402.

## Conflict of interest

The authors declare that the research was conducted in the absence of any commercial or financial relationships that could be construed as a potential conflict of interest.

## Publisher’s note

All claims expressed in this article are solely those of the authors and do not necessarily represent those of their affiliated organizations, or those of the publisher, the editors and the reviewers. Any product that may be evaluated in this article, or claim that may be made by its manufacturer, is not guaranteed or endorsed by the publisher.

## Footnotes

^1^We realized after completing our search that our search criteria, which focused on abstracts, had excluded many book chapters because they often do not include abstracts.

^2^During the process of addressing reviewer comments, the categorization of reasons for excluding articles was minorly revised to increase clarity and excluded articles were recategorized by the first author. This particular change did not impact whether articles were included or excluded; rather it changed the criteria and labels for different reasons for exclusion.

^3^Outcomes in this category were only found in the correlational (not experimental) studies.

^4^We do not conduct formal mediation analyses, calculate confidence intervals, or test significance here as it is beyond the scope of the present meta-analysis.

^5^The effect size in our work is similar in magnitude to those found by another meta-analysis examining the effect of gain and loss frame messages on positive (*d* = 0.31) and negative (*d* = 0.22) emotions (Nabi et al., 2020).
