# Supplementary material for: Hopium or empowering hope? A meta-analysis of hope and climate engagement
Source: Front Psychol. 2023 Aug 15;14:1139427. doi: 10.3389/fpsyg.2023.1139427 (PMC10465179; doi:10.3389/fpsyg.2023.1139427)
Supplement: Supplementary file 1 [file Data_Sheet_1.docx]

# Supplemental Analyses

## Interaction between Type of Behavior and Type of Study

We compare correlational and experimental studies to consider whether there is any evidence that action could be influencing hope in addition to (or possibly instead of) hope influencing action. Typically, many experimental manipulations more strongly predict behavioral intentions than self-reported or measured behavior (e.g., Maki et al., 2019) and the same is true for predictor variables in correlational studies. Yet, behavioral intentions reflect behavior that could occur in the future (and thus could not directly influence hope in the present), while in nonexperimental designs self-reported and measured behavior typically reflects behavior that happened in the past (and thus could influence hope in the present). In contrast, in experimental designs, measured and self-reported behavior typically occurs after the hope-inducing manipulation. Thus, if behaviors are increasing hope, we might expect that experimental manipulations more strongly increase intentions than actual behavior, but for cross-sectional studies to show a stronger effect of actual (past) behavior than (future) intentions. Due to a great number of possible confounds, we note that the results of this test would provide at best weak evidence in support of or against this notion.

A model comparison test with all studies included suggested that a model including the interaction between type of study (experimental vs. correlational) and type of measure (e.g., intentions, self-report), as well as main effects of each, did not add useful information compared to a model only including type of study, LRT(3) = 0.58, *p* = .90. This test provides preliminary tentative evidence against the notion that behavior is a major source of hope in the correlational studies.

## Exploring Between-Manipulation Differences in Increases in Hope

In Figure S1, we present a forest plot of manipulation effects on hope for all experimental studies that measured hope after the experimental manipulation. Several experimental studies are missing because they did not measure hope or we were not able to obtain the effect size.

**Figure S1**

*Forest plot of manipulation effectiveness at increasing hope*


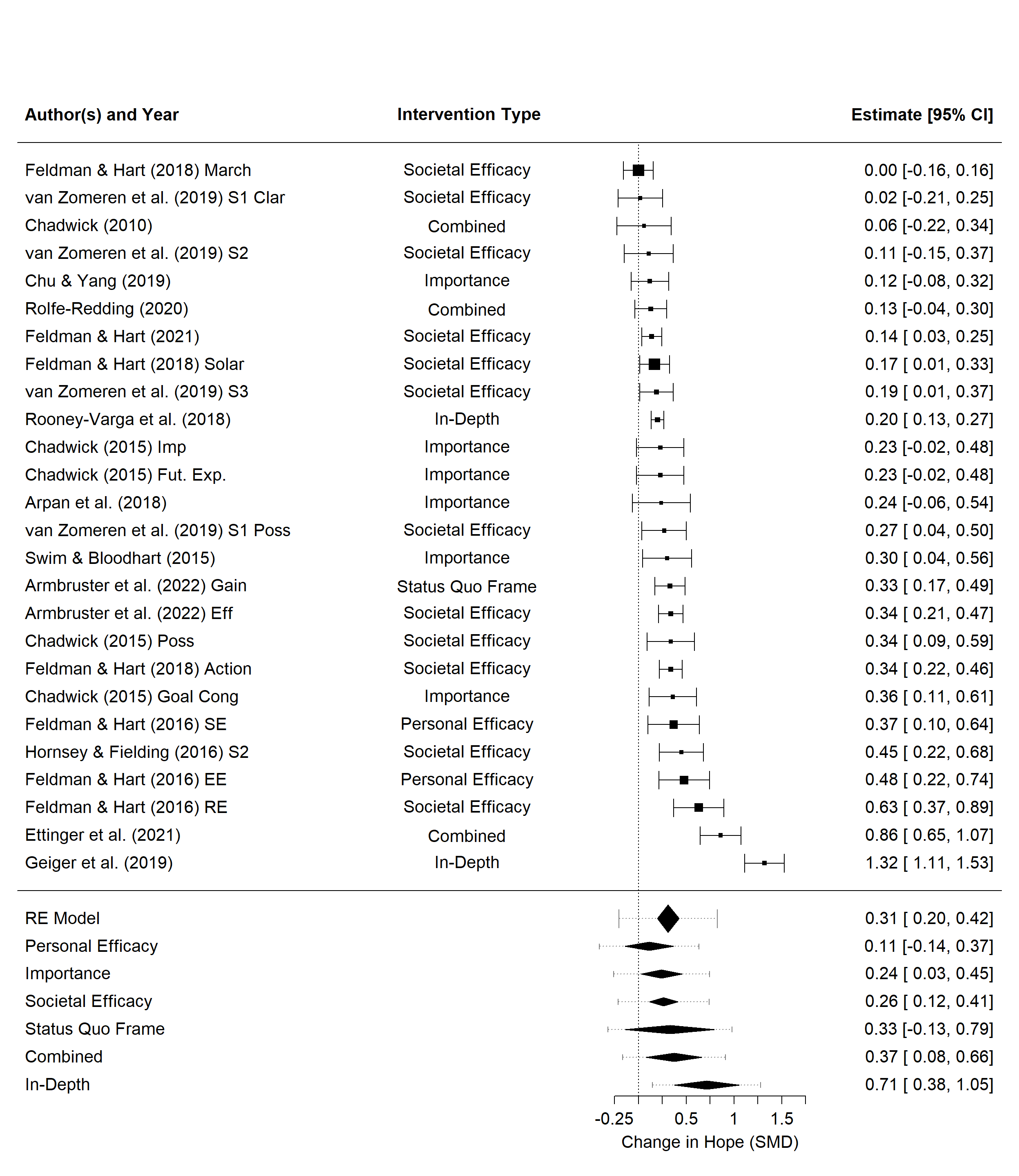


Note. For results by study, square sizes represent weights and solid lines represent confidence intervals. For summary statistics, diamonds represent confidence intervals and dashed lines represent prediction intervals.
SMD = Standardized Mean Difference between conditions (i.e., Cohen’s D).

## Supplemental Subgroup Analyses

Here we explore more complex subgroup analyses. In Table S1 we break down estimated effects by target of hope and type of engagement. In Table S2 we break down estimated effects by type of hope manipulation and type of engagement. We note that neither interaction is statistically significant (see main text), so we do not test differences here.

**Table S1**

Means and confidence intervals for correlations between each type of hope and each type of outcome.

|  | **Info-Seeking** | **Private** | **Public** | **Policy Support** | **Adapation** |
| --- | --- | --- | --- | --- | --- |
| **Denial** | NA | -.16 [-.35, .04]  (k = 2) | -.33 [-.49, -.15]  (k = 2) | -.42 [-.55, -.28]  (k = 3) | NA |
| **Climate Change** | -.10 [-.35, .17] (k = 1) | .18 [.03, .32]  (k = 4) | .08 [-.05, .21]  (k = 5) | .08 [-.05, .21]  (k = 6) | NA |
| **Domain-General** | NA | NA | NA | -.03 [-.30, .24]  (k = 1) | .28 [.04, .49]  (k = 2) |
| **Message** | NA | .23 [.08, .37]  (k = 5) | .28 [.08, .46]  (k = 2) | .26 [.08, .42]  (k = 3) | NA |
| **Goal** | NA | .31 [.16, .44]  (k = 4) | .21 [.11, .30]  (k = 10) | .31 [-.01, .58]  (k = 1) | NA |
| **Action** | NA | NA | .35 [.23, .47]  (k = 5) | .45 [.29, .58]  (k = 3) | NA |

Note. NAs reflect combinations that were not assessed in included work. Numbers outside brackets reflect mean estimates. Numbers in brackets reflect 95% confidence intervals.

**Table S2**

Means and confidence intervals for effects of each type of hope manipulation on each type of outcome.

|  | **Info-Seeking** | **Private** | **Public** | **Policy Support** |
| --- | --- | --- | --- | --- |
| **Status Quo Frame** | NA | -.99 [-1.66, -.32]  (k = 1) | NA | -.12 [-.29, .06]  (k = 2) |
| **Combined** | .00 [-.16, .15]  (k = 2) | .07 [-.05, .19]  (k = 6) | -.08 [-.27, .10]  (k = 2) | .03 [-.14, .21]  (k = 2) |
| **Societal Efficacy** | NA | .09 [-.12, .30] | .01 [-.09, .10]  (k = 5) | .09 [-.01, .18]  (k = 3) |
| **Importance** | NA | .03 [-.11, .18]  (k = 2) | .33 [.02, .64]  (k = 1) | .19 [-.01, .39]  (k = 2) |
| **Personal Efficacy** | NA | NA | .15 [-.04, .34]  (k = 1) | NA |
| **In-Depth** | NA | NA | .47 [.31, .62]  (k = 2) | NA |

Note. NAs reflect combinations that were not assessed in included work. Numbers outside brackets reflect mean estimates. Numbers in brackets reflect 95% confidence intervals.
